# Multi-target regulatory effects of rhaponticin in a rat model of hepatic fibrosis revealed by non-targeted metabolomics

**DOI:** 10.3389/fphar.2024.1505309

**Published:** 2025-01-14

**Authors:** Min Yang, Dihua Jiang, Longfei Huang, Tao Zhang, Wenfen Guo, Wenyan Lin, Jiali Zhao, Yunsheng Wei, Lang Peng, Yong-Jia Hao, Ying Zhou

**Affiliations:** School of Pharmacy, Guizhou University of Traditional Chinese Medicine, Guiyang, China

**Keywords:** hepatic fibrosis, traditional Chinese medicine, rhaponticin, metabolomic, molecular mechanism

## Abstract

**Introduction:**

Hepatic fibrosis (HF), a progressive chronic liver disease, is a serious threat to global public health. The lack of preventive and therapeutic strategies has created an urgent need for effective anti-fibrosis agents. There is growing evidence that natural products might provide safe and effective interventions for HF. Among them, rhaponticin (RHA), a stilbenoid glucoside natural product isolated from medicinal plants of *Rheum* L. of *Polygonaceae* Juss. has many pharmacological activities such as anti-inflammatory, antioxidant, antiproliferative, and antithrombotic properties. However, its effects on HF remain unclear.

**Methods:**

Herein, we investigated the effects of RHA against HF on the carbon tetrachloride (CCl_4_)-induced hepatic fibrosis and the underlying mechanism in rats. Functional, histopathological, and protein-level indicators of liver insult were evaluated. Moreover, serum metabolites were assessed by non-targeted metabolomics.

**Results and discussion:**

The results showed that RHA improved liver functions and histopathological features in the liver of CCl_4_-treated rats, and alleviated the expression of α-SMA and type I collagen. Meanwhile, RHA also modulated endogenous metabolite levels in rats with HF, targeting glycerophospholipid metabolism signaling and other pathways. These findings confirmed the protective effects of RHA against hepatic fibrosis in rats by exerting multi-target effects via multiple signaling and metabolic pathways. Which may be of use in developing more effective RHA-based therapeutic strategies for hepatic fibrosis.

## 1 Introduction

Hepatic fibrosis (HF) is a tissue response to long-term chronic liver insult caused by various factors, including chronic viral hepatitis, excess alcohol intake, metabolic dysfunction-associated steatohepatitis (MASH), and autoimmune diseases. It is characterized by massive deposition of diffuse extracellular matrix and abnormal hyperplasia of connective tissue (Karsdal et al., 2020; Villesen et al., 2020). Sustained hepatic damage will lead to progressive fibrosis and ultimately to cirrhosis and hepatocellular carcinoma, which is one of the main causes of morbidity and mortality worldwide (Dhanasekaran et al., 2019). HF represents a significant global health burden. Although researchers have made vast efforts to treat HF, effective preventive and therapeutic strategies are currently not available for many patients (Tan et al., 2021).

The histological features of HF vary with different etiologies (Villesen et al., 2020). Its mechanisms are complex, involving hepatic stellate cells (HSCs) activation (Ferdek et al., 2022), macrophages (Kazankov et al., 2019; Chen et al., 2022), hepatic sinusoidal endothelial cells (Du and Wang, 2022), inflammatory vesicles, pyroptosis, and mesenchymal stem cells (Wang et al., 2010; Gaul et al., 2021). Recent studies have revealed that the induction of lactate production by hexokinase 2 promotes histone lactylation, thereby regulating HF caused by HSCs activation. The inhibition of histone lactylation through HSC-specific or systemic deletion of hexokinase 2 effectively suppresses HSCs activation and hepatic fibrosis *in vivo*, highlighting a promising therapeutic target for the treatment of HF (Rho et al., 2023). Nonetheless, the mechanism of HF is very intricate. The interactions including various cells, chemokines, and signal pathways, form a huge network with mutual influences and feedbacks, make clinical treatments challenging. Thus, there is an urgent need to develop safe, effective, and affordable therapeutic strategies for hepatic fibrosis (Abraldes et al., 2016).

There is growing evidence that natural products isolated from traditional Chinese medicines could alleviate hepatic fibrosis, such as curcumin (Kheradpezhouh et al., 2016), puerarin (Li et al., 2018), and glycyrrhizin (Wang et al., 2017) and so on. With the achievements of natural products in the fight against hepatic fibrosis, understanding molecular mechanisms that underly their anti-fibrotic effects are critical for developing more effective therapeutic methods. Rhaponticin (RHA, 3,3′,5-trihydroxy-4′-methoxystilbene 3-O-β-D-glucoside, C_21_H_24_O_9_, Figure 2), a stilbenoid glucoside metabolite, can be found in medicinal plants of *Rheum* L. of *Polygonaceae* Juss. Such as *Rheum officinale* Baill., *Rheum rhabarbarum* L., *Rheum hotaoense* C. Y. Cheng and T. C. Kao., and *Rheum palmatum* L. (Chen et al., 2020). RHA exerted a wide range of pharmacological activities, including anti-inflammatory, antioxidant, antitumor, and antithrombotic properties (Park et al., 2002; Sun et al., 2012; Liang et al., 2013). Especially the inflammation and oxidative stress, which have long been considered to be responsible for the development and progression of hepatic fibrosis. RHA has showed significant anti-inflammatory effects, which attenuated intestinal damage *in vivo* and *in vitro* associated with the response of reducing inflammation (Wei et al., 2017). In addition, RHA demonstrated antioxidant potential, which could as a powerful potent oxygen radical scavenger to exert protective effects (Zhang et al., 2007).

In this context, simultaneously considering that preventing and treating hepatic damage is one of research interests in our group (He et al., 2019; Liu et al., 2021), we postulated that RHA could potentially be of use in intervening HF, and the mechanism has not been investigated to date. Accordingly, the present study was conducted to explore the anti-fibrotic effects of RHA in a rat model of HF induced by carbon tetrachloride (CCl_4_). In animals, many causes of fibrogenesis have been researched. While intraperitoneal injection of CCl_4_ is a widely used method for the establishment of hepatic fibrotic models to simulate the pathogenesis of human hepatic fibrosis. CCl_4_ is a hepatotoxin, whose mechanism is mainly oxidative damage caused by lipid peroxidation. Specifically, cytochrome P450 enzyme can convert CCl_4_ into highly reactive trichloromethyl radical (·CCl_3_), ultimately leading to hepatotoxic damage, inflammation and fibrosis (Bao et al., 2021).

As expected, our results demonstrate that RHA exerts anti-fibrotic effects on the liver by targeting multiple signaling and metabolic pathways, which not only identifies RHA as a promising anti-fibrotic lead compound for further research, but also provides a reference for developing effective anti-fibrotic agents.

## 2 Materials and methods

### 2.1 Materials

Rhapontin (RHA, batch number: PRF22042144, purity over 98%) was purchased from Chengdu Biopurify Phytochemical Ltd. (Chengdu, China). Colchicine Tablets (batch number: 41,632) were manufactured by Yunnan Phytopharmaceutical Co., Ltd. (Kunming, China). Dimethyl sulfoxide (DMSO, batch number: 22030130) was procured from Chengdu Jinshan Chemical Reagent Co., Ltd. (Chengdu, China). Carbon tetrachloride (CCl_4_, batch number: 20190428) was sourced from Chongqing Jiangchuan Chemical Co., Ltd. (Chongqing, China). Kits for AST, ALT, HYP and ELISA Kits for Col-IV, LN and HA were obtained from Nanjing Jiancheng Bioengineering Institute (Nanjing, China). Antibody for α-SMA (Cat No. Ab5694) was obtained from Abcam (Cambridge, MA, United States). Antibody for Col-Ⅰ (Cat No. GB11022-3) and secondary antibody for Goat anti-rabbit (Cat No. GB23303) were purchased from Wuhan Servicebio Technology Co., Ltd. (Wuhan, China). The rat HSC line HSC-T6 and the human HSC line LX-2 cells were obtained from China center for type culture collection (Wuhan, China), RPMI-1640, FBS and penicillin streptomycin were purchased from Procell Life Science &Technology Co., Ltd. (Wuhan, China). Cell Counting Kit-8 (CCK-8) were obtained from UElandy Inc. (Suzhou, China).

### 2.2 Preparation of RHA solutions

RHA was dissolved in DMSO and prepared into 200 mM stock solution, which was completely dissolved after standing in a warm water bath at 37°C for 5 min. The cell activity assay was performed using a gradient dilution method with 0–150 μM RHA.

### 2.3 Cell culture

The rat HSC line HSC-T6 and the human HSC line LX-2 were cultured in RPMI-1640 medium supplemented with 10% fetal bovine serum (FBS), 100 U/mL penicillin and 100 U/mL streptomycin in a 37°C, 5% CO_2_, saturated humidity environment.

### 2.4 CCK-8 determination

HSC-T6 and LX-2 cells (1 × 10^4^) were seeded in a 96-well plate and incubated overnight, and then different concentrations of RHA (0–150 μM) were added. Then, the cells were incubated for 48 h, and 100 μL of 10% Cell Counting Kit-8 (CCK-8) assay reagents were added to each well. The optical density (OD) value was measured at 450 nm using a spectrophotometer.

### 2.5 Animals and treatments

The procedures for this study were approved by Guizhou University of Traditional Chinese Medicine (No. 20230096). All animal experiments in this study were conducted in accordance with the guidelines for ethical review of animal welfare in China (GB/T 35892-2018, the State Standard of the People’s Republic of China). All methods were carried out in accordance with relevant guidelines and regulations. The thirty male SD rats (8 weeks-old), weighing 180–220 g, were provided from the Institute of Laboratory Animal Science of Guizhou University of Traditional Chinese Medicine (Guiyang, China). All rats were housed under the controlled temperature (20–25°C) and on a 12 h light and 12 h dark cycle with food and water *ad libitum*. All rats were adapted to their new housing conditions for 1 week before the experiments. The rats were randomly divided into 5 groups (n = 6 in each group) as follows: control group, CCl_4_-treated model group, colchicine-treated group, high (25 mg/kg) and low dose (6.25 mg/kg) of RHA-treated groups. CCl_4_-treated rats were injected intraperitoneally with 50% CCl_4_ (1 mL/kg) diluted in olive oil every other day for 4 weeks. From the 5th week, rats were dosed once daily by oral gavage with either RHA or same volume of vehicle for 21 days. At the end of the experiment, cardiac blood and liver tissues of rats were collected.

### 2.6 Liver histopathology

The harvested liver tissues were fixed in 4% formalin, embedded in paraffin, sectioned at 5 μm and stained with haematoxylin–eosin (HE), Masson trichrome according to standard procedures. The morphologies were observed with a microscope (Panthera, China).

### 2.7 Biochemical detections

The contents of AST, ALT, HYP, HA, Col IV and LN were assayed with microplate detection kits following the standard instructions, and read using Thermo Scientific spectrophotometer.

### 2.8 Immunohistochemistry analysis

The paraffin sections (5 μm thick) were incubated with 3% H_2_O_2_ to eliminate endogenous peroxidase. When non-specific binding sites were blocked by goat serum for 20 min at room temperature, then incubated with α-SMA antibody and Collagen I antibody overnight at 4°C. And second antibodies conjugated with HRP at 37°C for 30 min. The paraffin sections were incubated with DAB for 2 min, counterstained with hematoxylin for 3 min, and counterstained with blue reagent for 10 s, then viewed with a Motic BA410 microscope at ×400 magnification.

### 2.9 Metabolomic analysis

100 μL liquid sample was added to a 1.5 mL centrifuge tube with 300 μL solution (acetonitrile: methanol = 1:1, v/v) containing 0.02 mg/mL internal standard (L-2-chlorophenylalanine) to extract metabolites. The samples were mixed by vortex for 30 s and sonicated for 30 min at 5°C (40 KHz). The samples were placed at −20°C for 30 min to precipitate the proteins. Then the samples were centrifuged for 15 min (4°C, 13,000 g). The supernatant was removed and blown dry under nitrogen. The sample was then re-solubilized under the above conditions, followed by centrifugation for 10 min (4°C, 13,000 g). Finally, the supernatant was transferred to sample vials for LC-MS/MS analysis. Quality control sample, UHPLC-MS/MS analysis, and statistical analysis were provided in the Supporting Information.

## 3 Results

### 3.1 RHA inhibits the proliferation of activated HSCs

**FIGURE 1:**
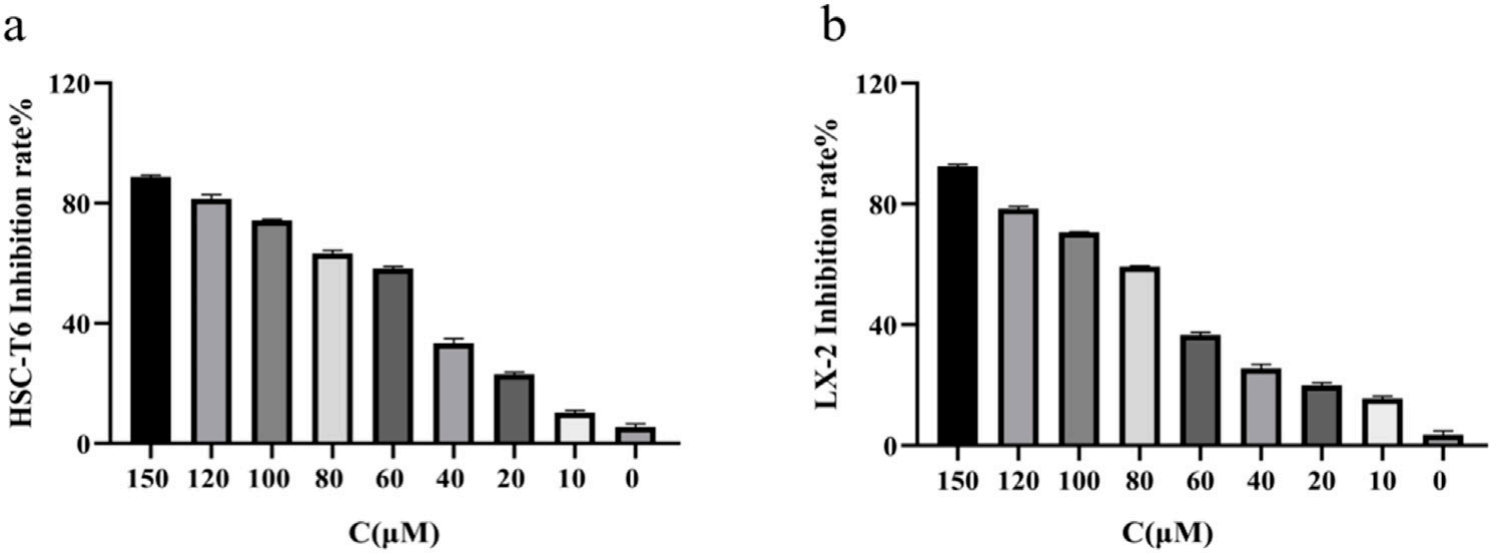
Effect of RHA on the proliferation of HSC-T6 and LX-2 cells. **(A)** Effect of RHA on the proliferation of HSC-T6 cells using the CCK8 assay. **(B)** Effect of RHA on the proliferation of LX-2 cells using the CCK8 assay. The results are presented as means ± SD.

To evaluate the effects of RHA on HSCs proliferation, the CCK-8 assay was used to detect the effects of 48-h treatment with 0–150 μM RHA on activated HSC-T6 (Figure 1A) and LX-2 (Figure 1B) cells. The IC_50_ values of RHA in HSC-T6 and LX-2 cells were 73 and 87 μM, respectively. These results suggest that RHA can inhibit the proliferation of activated HSC-T6 and LX-2 cells in a dose-dependent manner, providing preliminary evidence that RHA inhibits HSCs activation and may inhibit the development of hepatic fibrosis. Therefore, next, we investigated this postulation *in vivo* using SD rats.

### 3.2 RHA alleviates pathological liver damage in CCl_4_-Induced rats

**FIGURE 2:**
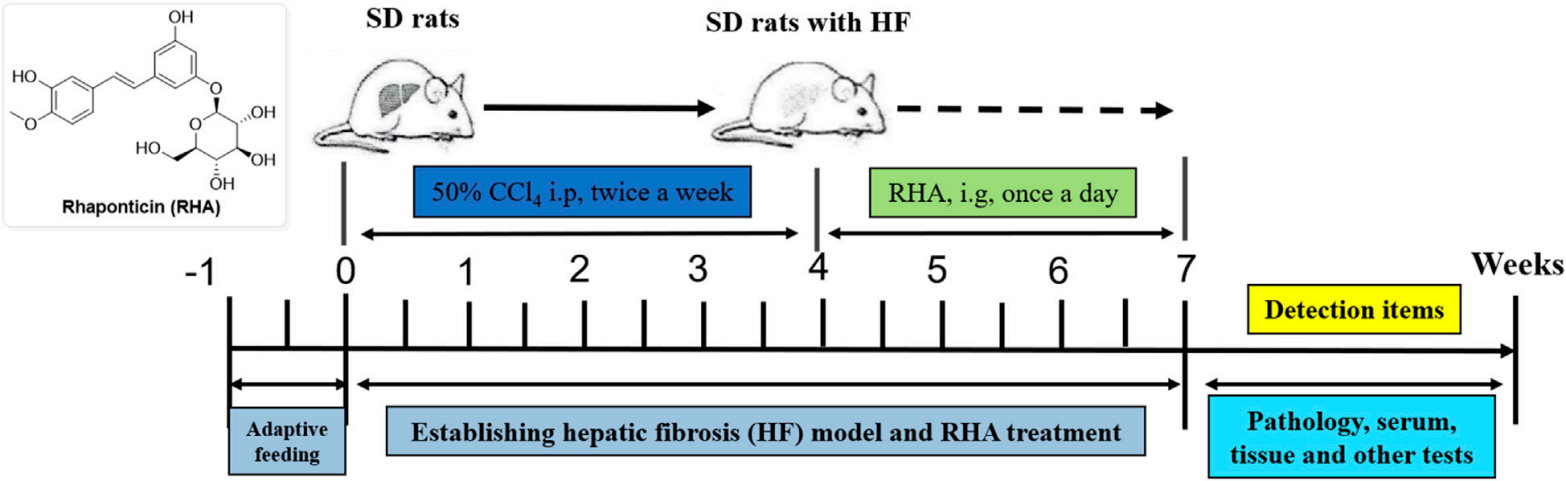
Schematic of the animal experiment.

**FIGURE 3:**
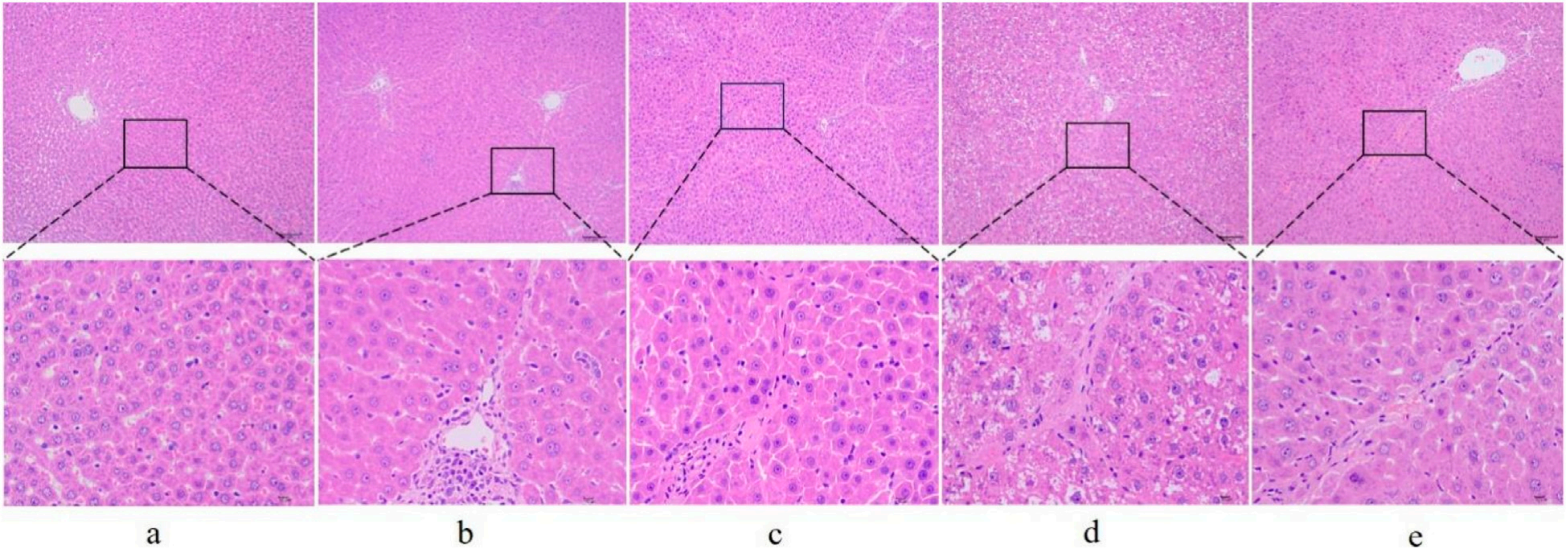
Representative images of liver sections from different treatment groups stained with H&E (original magnification: ×100 and 400×, scale bar: 100 μm). **(A)** Control group; **(B)** model (CCl_4_-treated) group; **(C)** colchicine group; **(D)** low dose RHA (6.25 mg/kg); **(E)** high dose RHA (25 mg/kg).

**FIGURE 4:**
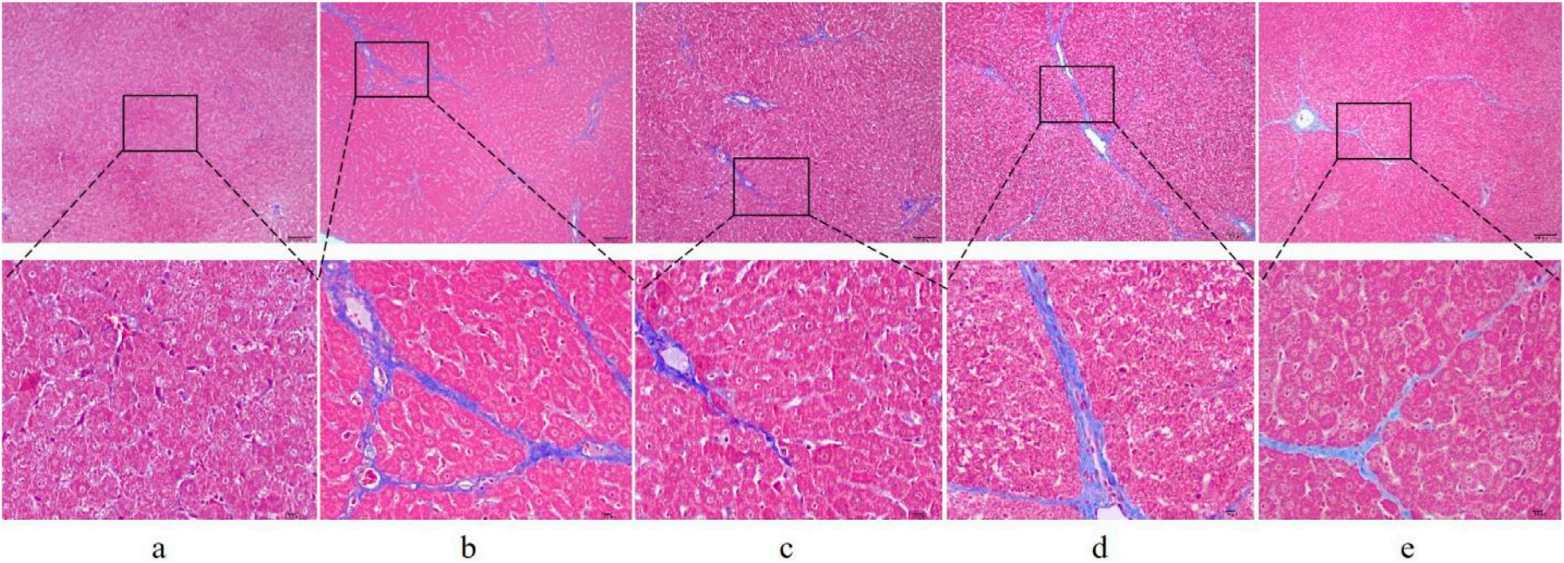
Representative images of liver sections from different treatment groups stained with Masson’s trichrome stain (original magnification: ×100 and 400×; scale bar: 100 μm). **(A)** Control group; **(B)** model (CCl_4_-treated) group; **(C)** colchicine group; **(D)** low dose RHA (6.25 mg/kg); **(E)** high dose RHA (25 mg/kg).

First, to investigate whether RHA has an antifibrotic effect *in vivo*, the experimental model was established. The overall process of the experiment is shown in Figure 2. Thirty male SD rats were randomly divided into five groups (n = 6), injected with 50% CCl_4_ solution for 4 weeks (Dong et al., 2018), and orally administered with RHA (25 or 6.25 mg/kg/day) every day from the fifth week for 21 days. At the end of the experiment, hematoxylin and eosin (H&E) and Masson’s trichrome staining were performed to determine the pathology of hepatic fibrosis. As shown in Figure 3, H&E staining demonstrated that the liver lobular structures were normal with central veins and radial hepatic cords in the control group (Figure 3A). However, in the CCl_4_-treated group, the liver tissue exhibited a mass of inflammatory cell infiltrates and a disordered arrangement of liver cells (Figure 3B). In contrast, RHA and colchicine notably inhibited these pathological changes (Figures 3C–E). Moreover, Masson’s trichrome staining demonstrated that hepatic fibrosis was successfully established (Figure 4B). RHA treatment could alleviate the collagen deposition caused by CCl_4_ *in vivo* (Figures 4D, E). Taken together, these data suggest that RHA attenuates HF and improves liver functions in CCl_4_-treated rats.

### 3.3 RHA attenuates CCl_4_-Induced HF in rats

**FIGURE 5:**
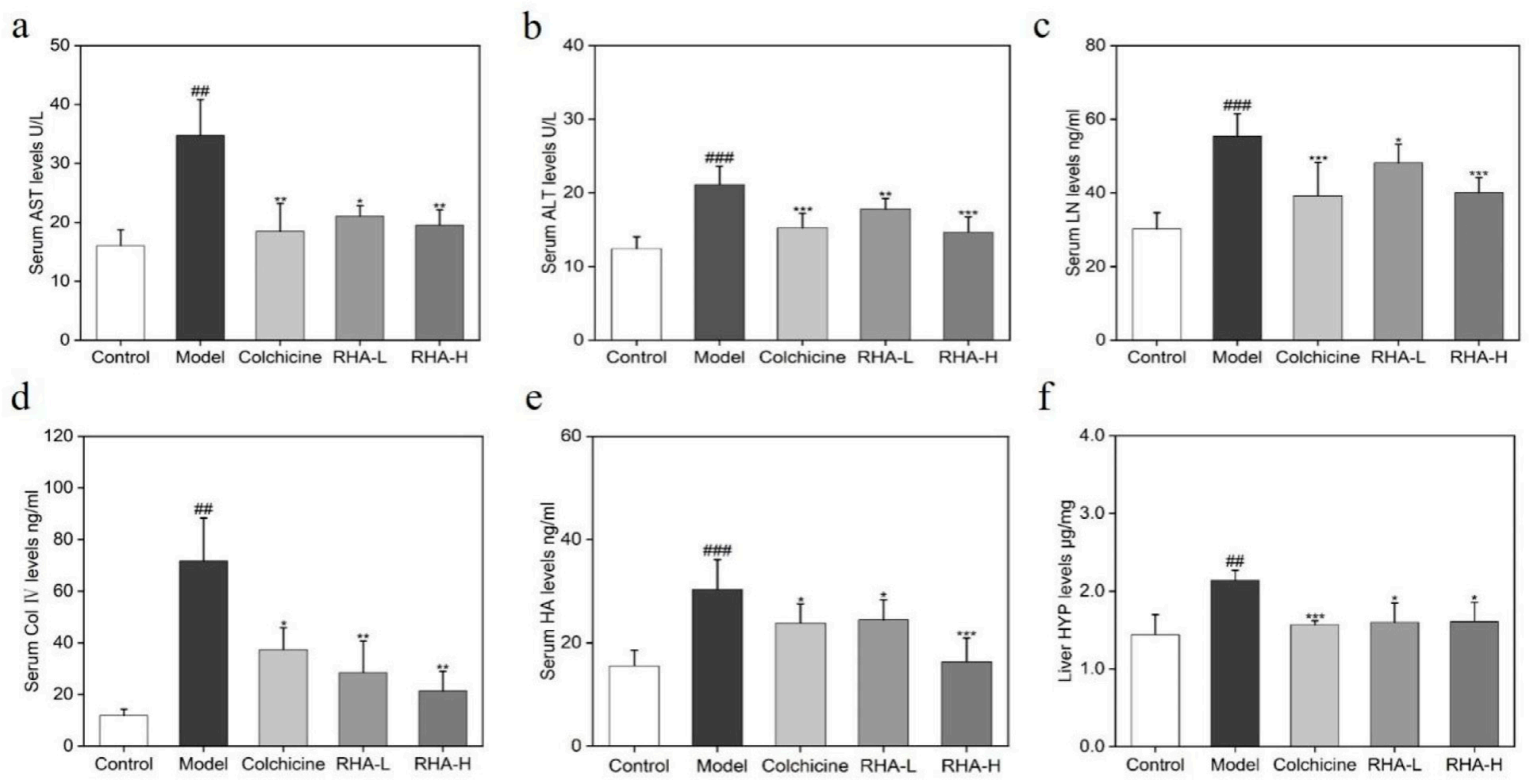
RHA attenuates liver injury induced by CCl_4_ in rats. Serum levels of **(A)** AST; **(B)** ALT; **(C)** LN; **(D)** Col Ⅳ; **(E)** HA; **(F)** Liver HYP levels. RHA-L, low dose RHA (6.25 mg/kg); RHA-H, high dose RHA (25 mg/kg). Data are presented as means ± SD (n = 6). ^#^p < 0.05, ^##^p < 0.01, and ^###^p < 0.001, compared with the control group. ^*^p < 0.05, ^**^p < 0.01 and ^***^p < 0.001, compared with the model (CCl_4_-induced) group.

Next, indicators of liver functions and liver fibrosis were examined. The serum levels of aspartate transaminase (AST), alanine transaminase (ALT), hyaluronic acid (HA), collagen (Col) IV, laminin (LN), and liver hydroxyproline (HYP) were measured. Specifically, AST and ALT are two key liver enzymes that reflect hepatocyte integrity (Giannini et al., 2005; Kubes and Jenne, 2018). It can be seen from Figures 5A, B that RHA regulated AST and ALT levels in the rat model.

HYP, HA, LN, and Col IV are important biomarkers of HF, and their elevated levels are the main markers of HF and progression to cirrhosis (Zhang et al., 2016; Kim et al., 2017; Kong et al., 2019). In this study, compared with the control group, serum HA, LN, Col IV, and liver HYP levels increased markedly in the model group (p < 0.01), whereas RHA-treated groups reduced these levels (p < 0.01). Overall, the above observations suggest that RHA has potential hepatoprotective efficacy.

### 3.4 RHA affects protein levels of alpha smooth muscle actin (α-SMA) and collagen I (Col I) in a rat model of HF

**FIGURE 6:**
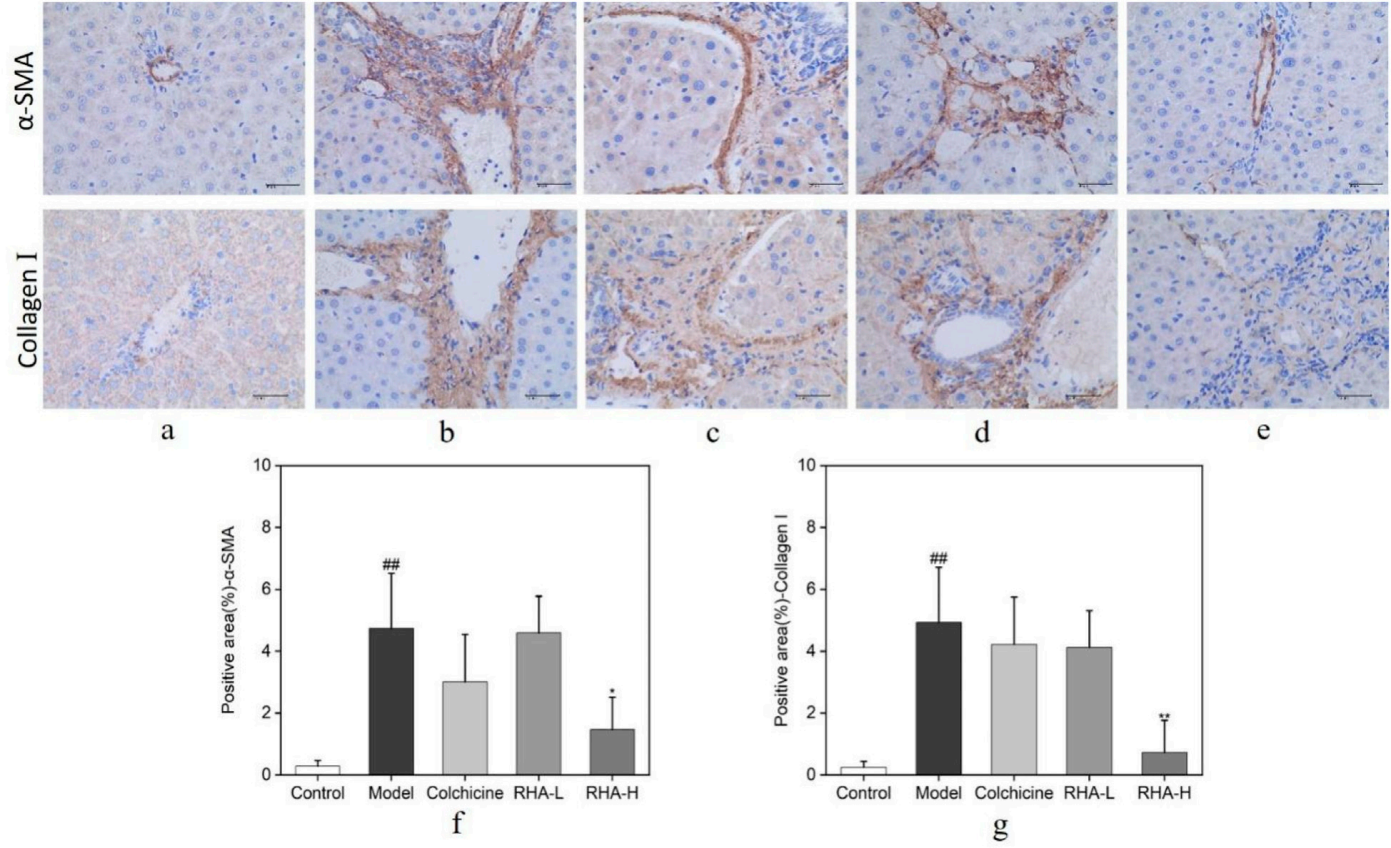
Protein levels of α-SMA and Col I, analyzed using immunohistochemical staining. Brown indicates positive staining (original magnification: ×400; scale bar: 100 μm). RHA-L, low dose RHA (6.25 mg/kg); RHA-H, high dose RHA (25 mg/kg). **(A)** Control group; **(B)** model group; **(C)** colchicine group; **(D)** low dose RHA (6.25 mg/kg); **(E)** high dose RHA (25 mg/kg); **(F)** The expression level of α-SMA in the liver; **(G)** The expression level of Col I in the liver; Data are presented as the mean ± SD (n = 6). ^#^p < 0.05 and ^##^p < 0.01, compared with the control group. *p < 0.05 and **p < 0.01, compared with the model (CCl_4_-induced) group.

We explored the effects of RHA on α-SMA and Col I protein levels in rat liver tissues using immunohistochemistry. As shown in Figure 6, compared with the control group, the cells in the model (CCl_4_-induced) group appeared plump. Most of the nuclei were stained blue, and a large number of α-SMA and Col I deposits was seen in the confluent area, central venous wall, and fibrous intervals (Figure 6B). Relative to the model group, the expression of α-SMA and Col I were reduced to different degrees in the RHA-treated groups, and the staining of the cell nuclei became lighter (Figures 6D, E). We also observed that RHA reduced α-SMA and Col I protein levels in hepatic tissue in the rat model. Herein, RHA significantly decreased the hepatic levels of α-SMA and Col I, suggesting that it could effectively ameliorate HF in the rat model.

### 3.5 Serum metabolic profiles

**FIGURE 7:**
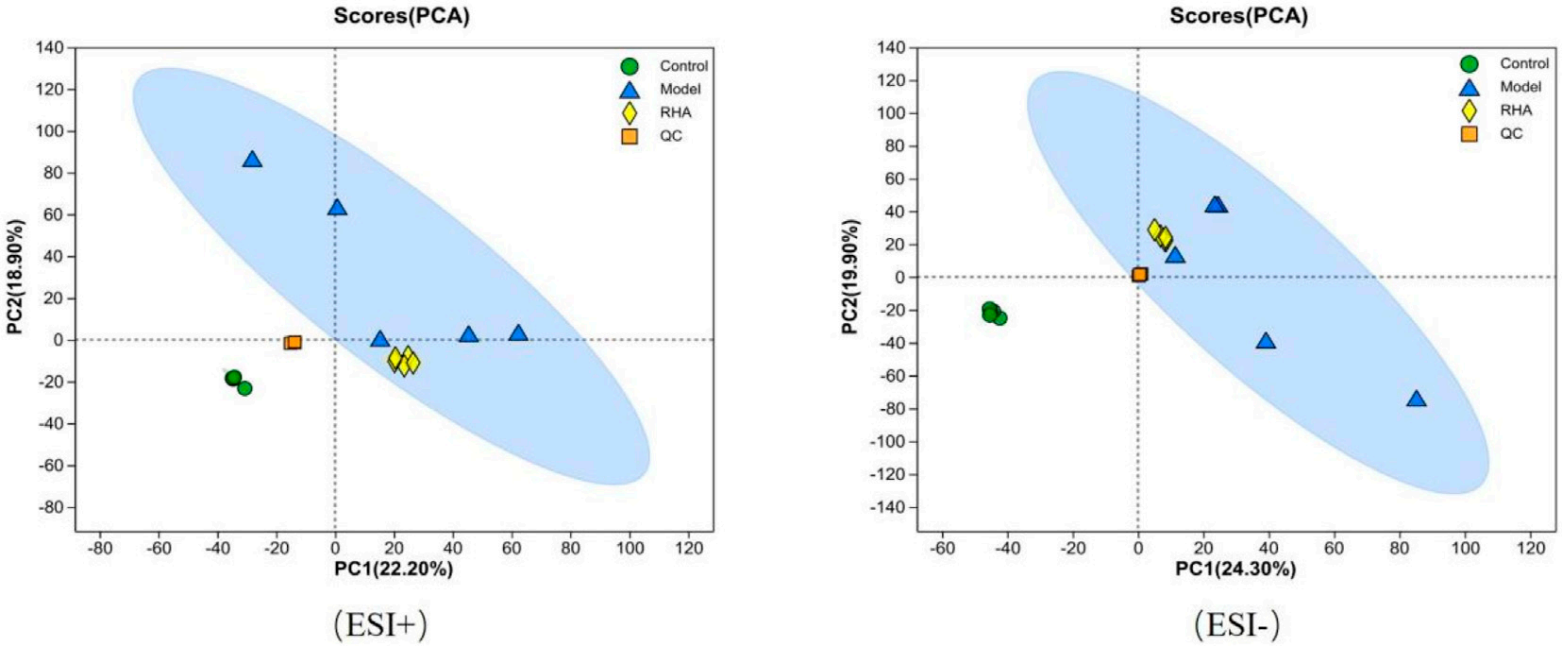
PCA score plot based on UPLC-MS/MS data for serum samples from the control, model (CCl_4_-induced), and RHA treatment groups, and QC samples.

UPLC-MS/MS (Ultra Performance Liquid Chromatography Tandem Mass Spectrometry) serum metabolomics analysis revealed the different metabolomics features in the three groups. Both Principal Component Analysis (PCA) and Orthogonal Partial Least Squares-Discriminant Analysis (OPLS-DA) score plots showed that there were significant differences between the control, model (CCl_4_-induced), and RHA-treated groups, indicating that different statuses have different serum metabolomics profiles (Figure 7).

The PCA plots for the control, model, RHA-treated groups, and quality control (QC) samples are shown in Figure 7. PCA of data obtained in the positive (ESI+) and negative (ESI–) ion modes revealed that the QC samples were tightly clustered, indicating high sequencing reproducibility to ensure data reliability. Data for each group (control, model, RHA-treated and QC) clustered with no significant overlap. We detected a significant difference in the metabolites between the control and model groups, indicating a significant change in the metabolite pattern in the latter. Similarly, significant changes in the metabolic profiles we observed between the model and RHA-treated groups (Figure 7).

**FIGURE 8:**
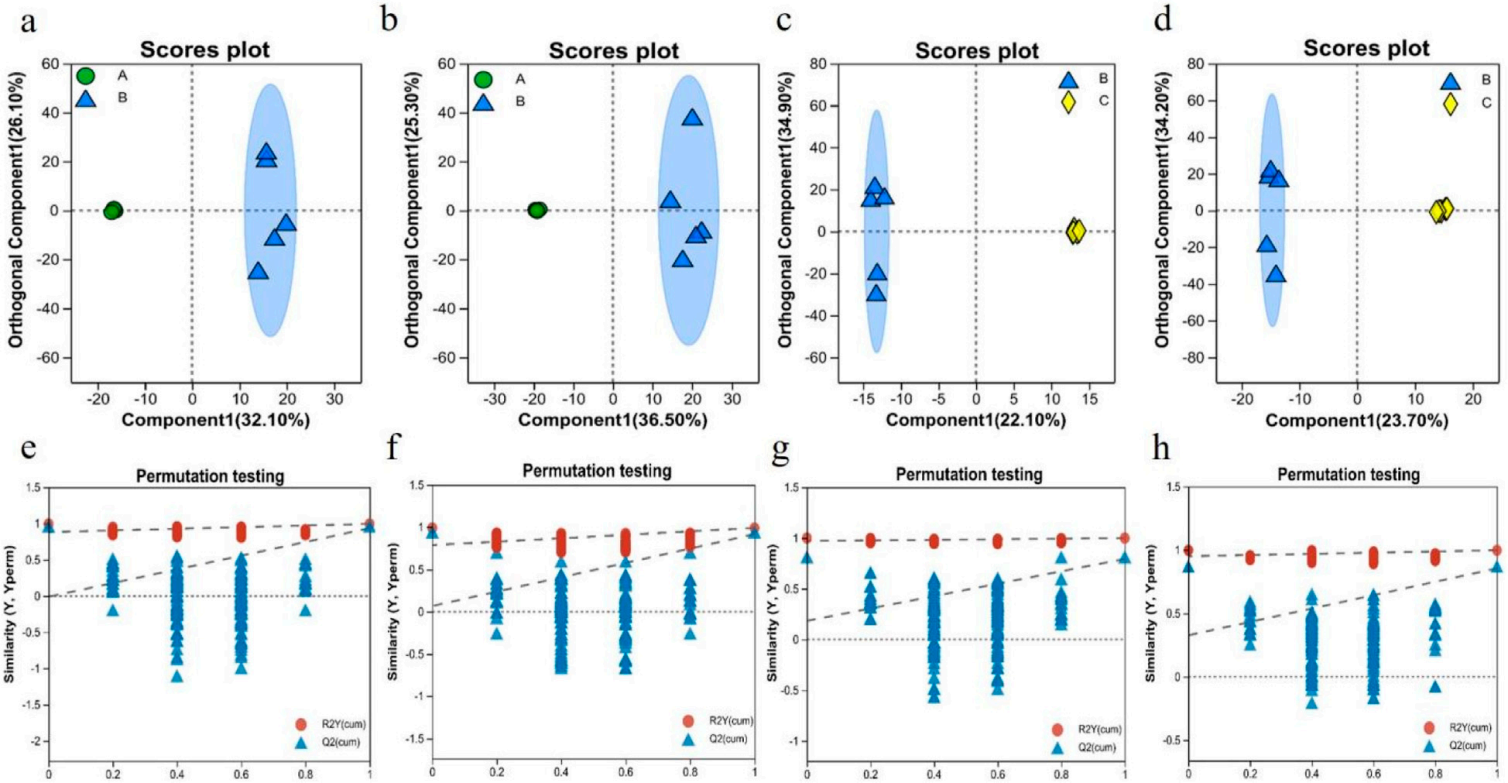
OPLS-DA score plots of serum metabolic profiles of different animal groups, acquired in the ESI + mode **(A, C)** and ESI–mode **(B, D)**, with the cross-validation plot of OPLS-DA model in the ESI + mode **(E, G)** and ESI–mode **(F, H)**. a, b, e, f, the control group vs. model (CCl_4_-induced) group; c, d, g, h, the model vs. RHA treatment group (n = 5).

The control, model, and RHA-treated groups were distinguished using OPLS-DA score plots. OPLS-DA is a supervised multivariate statistical approach for emphasizing logical distinctions between groups, while ignoring arbitrary differences within them. As shown in Figures 8A–D, serum samples from rats in the control and model groups were significantly separated in the plots, indicating a significantly altered serum metabolome of rats with hepatic fibrosis. The metabolic profiles of the model and RHA-treated groups were also significantly different, indicating that RHA impacts the disordered hepatic metabolism in CCl_4_-treated rats (Nuli et al., 2019).

The evaluation parameters of the OPLS-DA model were R^2^Y = 0.993 and Q^2^ = 0.934 in the ESI + mode, and R^2^Y = 0.989 and Q^2^ = 0.917 in the ESI–mode, for the control vs. model comparisons; and R^2^Y = 0.999 and Q^2^ = 0.794 in the ESI + mode, and R^2^Y = 0.998 and Q^2^ = 0.855 in the ESI–mode, for the model vs. RHA comparisons. Generally, the closer R^2^Y and Q^2^ are to 1, the more stable and reliable is the assessment model (Liang et al., 2019). Hence, the results indicate that the three assessment models (control, CCl_4_-induced, and RHA-treated groups) were valid, providing a good basis of the explanation and prediction of the differences among the groups (Figures 8E–H).

### 3.6 Classification of differential metabolites in serum samples

**FIGURE 9:**
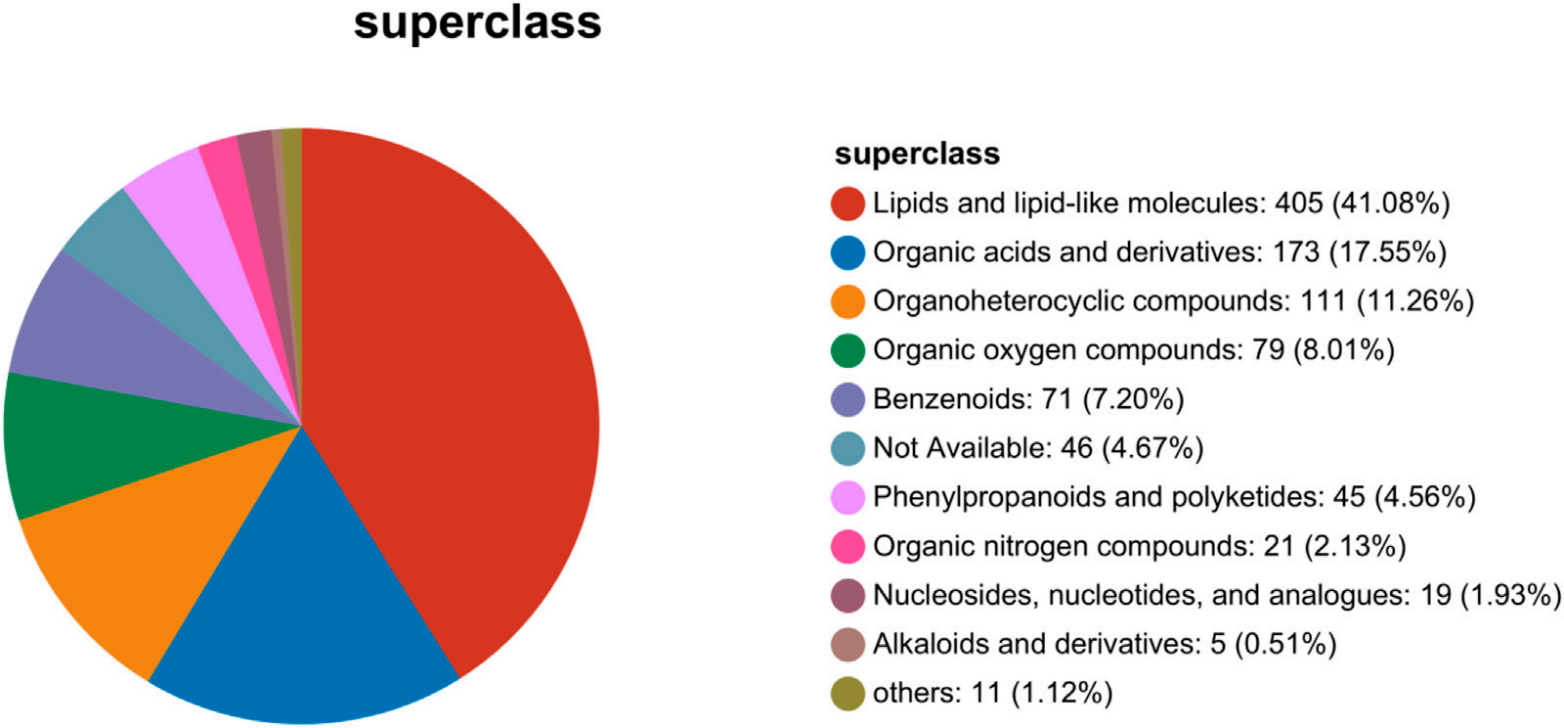
Statistics chart of Human Metabolome Database metabolites classification.

Based on metabolites classification in the Human Metabolome Database (HMDB Version 4.0), the detected differential metabolites were mainly lipids and lipid-like molecules, organic acids and derivatives, organoheterocyclic metabolites, organic oxygen metabolites, benzenoids, phenylpropanoids, and polyketides. Lipids and lipid-like molecules were the largest proportion (Figure 9). In pathological conditions such as hepatic fibrosis, disturbances in lipid metabolism may lead to the accumulation of fatty acids in the liver. This, in turn, might trigger oxidative stress, inflammatory responses, and hepatocellular damage. These processes not only exacerbate the procession of hepatic fibrosis, but may also affect the functions and metabolisms of other organs. Therefore, the high proportion of lipid and lipid-like molecules observed in this study may suggest significant changes in lipid metabolism during hepatic fibrosis.

### 3.7 Identification of differential metabolites in serum samples

**FIGURE 10:**
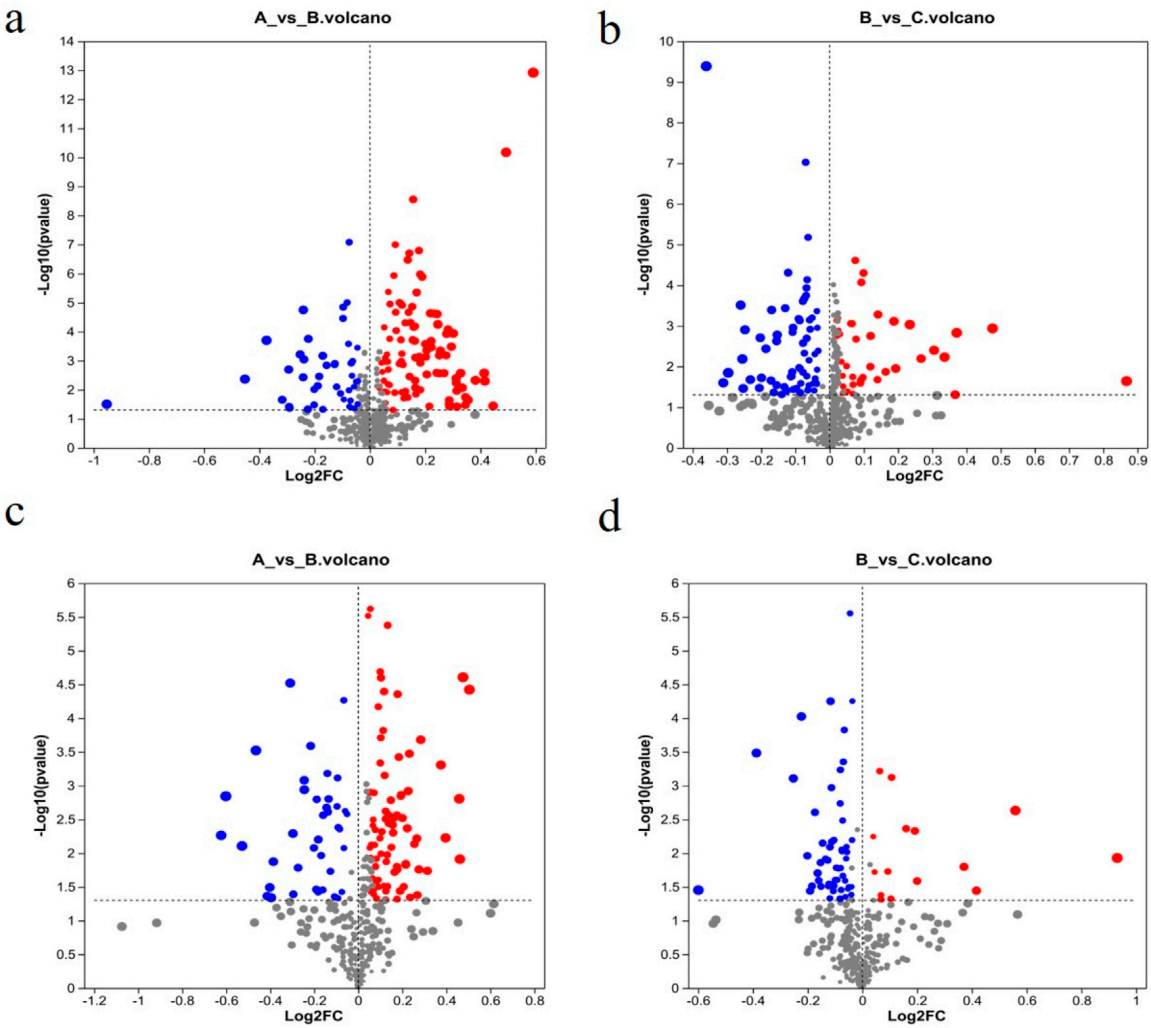
Volcano plots of metabolites from different groups. **(A)** Control group; **(B)** model group; **(C)** RHA-treated group. ESI + mode **(A, B)** and ESI–mode data **(C, D)**. Blue, downregulation; red, upregulation; gray: no significant change.

**FIGURE 11:**
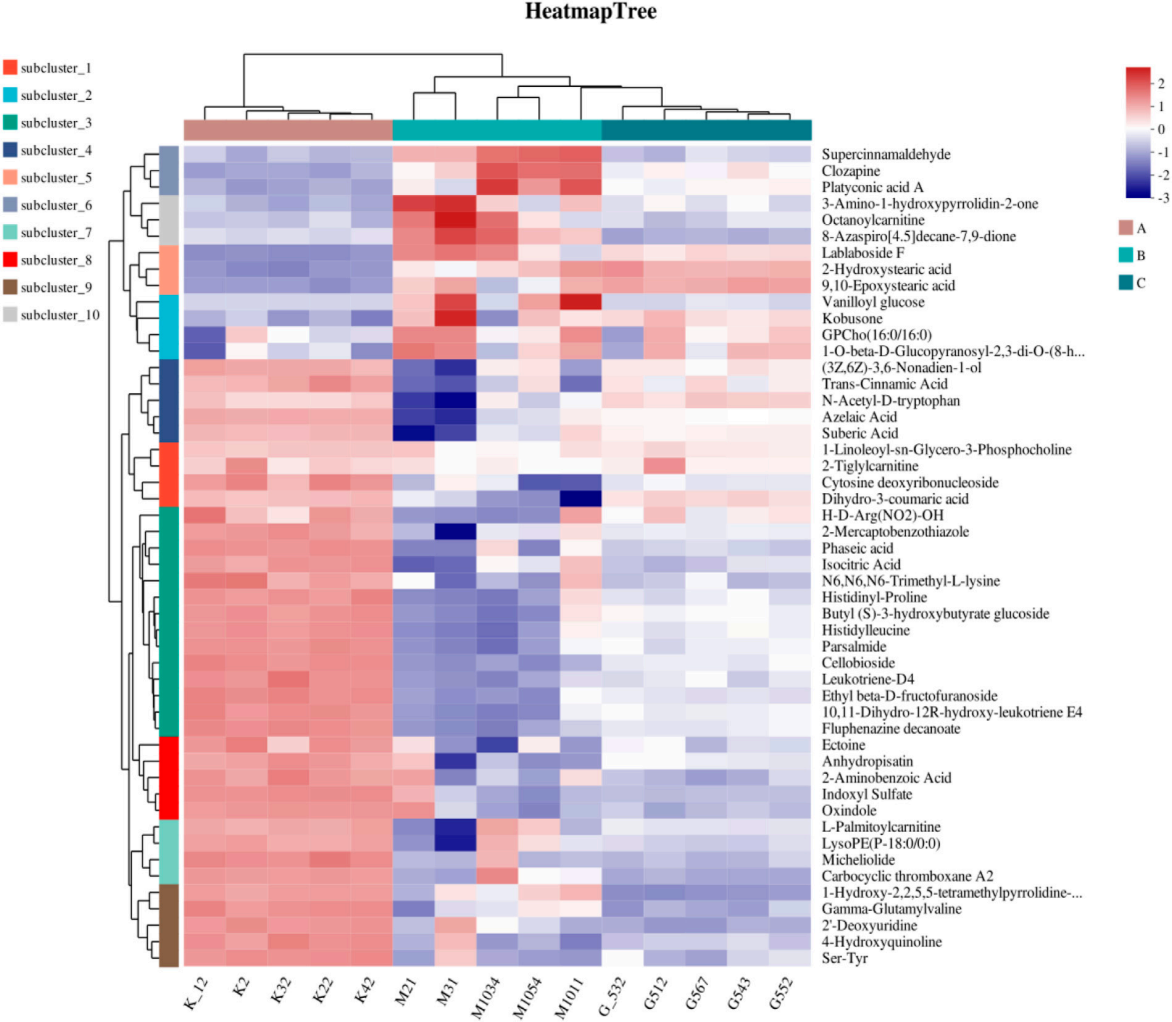
Heat maps of differential metabolites. (A) Control group (K12 to K42); (B) model group (M21 to M011); (C) RHA-treated group (G532 to G552).

Following the PCA and OPLS-DA, all metabolites detected in the ESI+ and ESI–modes were analyzed, and metabolite volcano plots were generated (Figure 10). Heat maps of metabolite abundance are shown in Figure 11, with red indicating high level and blue indicating low level.

Significantly differential metabolites were selected based on the criteria of the OPLS-DA model of variable importance in projection (VIP) score >l and p < 0.05. LC-MS metabolomics analysis revealed 267 differentially enriched metabolites between the control and model groups, 186 of the metabolites were elevated in the model, while 81 of them were decreased (see Supplementary Table 1 of the ESI). There were 173 metabolites that were significantly different between the model and RHA-treated groups (Supplementary Table 2), of which 54 were upregulated and 119 were downregulated in RHA-treated rats. The representative 25 metabolites are shown in Supplementary Tables 1, 2.

Further analysis of the screening results revealed that there were 80 differential metabolites that were common in the three groups (control vs. model group; model vs. RHA-treated group) that shared the opposite trend, among which 66 were upregulated and 14 were downregulated by RHA. The representative 25 differential metabolites are shown in Supplementary Table 3.

### 3.8 Metabolic pathway analysis of differential serum metabolites

**FIGURE 12:**
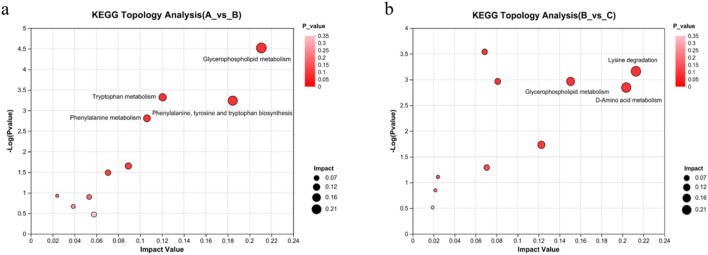
Effects of RHA on major metabolic pathways, as determined by metabolomic analyses of the serum in a hepatic fibrosis rat model. **(A)** Control vs. model (CCl_4_-induced) groups, glycerophospholipid metabolism; phenylalanine, tyrosine, and tryptophan biosynthesis; tryptophan metabolism; phenylalanine metabolism. **(B)** Model vs. RHA-treated group, lysine degradation, glycerophospholipid metabolism; d-amino acid metabolism.

Lastly, we used python packages ‘scipy.stats’ (https://docs.scipy.org/doc/scipy/) to perform enrichment analysis of the metabolomic data to identify biological pathways most relevant to the different experimental treatments. The analysis revealed 41 metabolic pathways involved in the 267 differential metabolites in the control group vs. the model group. These pathways were considered as potentially relevant when the pathway impact score >0.1 and–log_10_(p) > 2 (He et al., 2024). Four metabolic pathways satisfied these criteria, viz. glycerophospholipid metabolism; phenylalanine, tyrosine, and tryptophan biosynthesis; tryptophan metabolism; and phenylalanine metabolism (Figure 12A).

In addition, the 173 differential metabolites in the model vs. RHA-treated group comparison were involved in 44 metabolic pathways. Lysine degradation, d-amino acid metabolism, and glycerophospholipid metabolism were potentially relevant (Figure 12B), providing essential guidance for further research on molecular mechanism.

## 4 Discussion

Hepatic fibrosis plays an important role in the process of “chronic hepatitis–hepatic fibrosis–liver cirrhosis–liver cancer”. It is also a key risk factor for the development of liver cancer, a major disease characterized by high mortality worldwide. Due to the complex pathogenesis, no effective therapeutic agents are currently available. Alleviating or reversing HF is a significant approach to preventing liver cirrhosis and hepatocellular carcinoma, and developing effective and safe drugs is also the key to treat the disease.

Natural products have always been an important source of drug research and development, and some natural products could prevent or reverse the progression of HF. RHA is a stilbene natural product with a wide range of pharmacological activities such as anti-inflammatory, antioxidant, antitumor, hypolipidemic, and antithrombotic effects, and is a pioneering compound with development potential. Herein, this study aimed to explore the protective effects, and the underlying mechanisms of RHA against HF in a rat model. The results showed that RHA regulated AST and ALT levels in the rat model. The ALT and AST levels in the hepatic fibrosis model were recovered following RHA treatment, suggesting that RHA could protect hepatocytes from chronic injury. Furthermore, RHA significantly reduced the serum levels of HYP, Col IV, HA, and LN in rats with HF, indicating its efficacy in improving HF in rats owing to its good hepatoprotective effect. HSCs activation is well established as a central driver of fibrosis in liver injury. And, α-SMA is a marker of HSCs activation, while Col I is the main extracellular matrix metabolite released upon HSCs activation (Liu et al., 2019; Higashi et al., 2017; Yoneda et al., 2016). Therefore, detection of α-SMA expression can be used to assess the degree of hepatic fibrosis (Dong et al., 2018). Col I is a major metabolite of collagen in fibrotic liver tissue, and its hepatic levels can also reflect the degree of hepatic fibrosis (de Gouville et al., 2005). In this study, we observed that RHA significantly reduced α-SMA and Col I protein levels in hepatic tissue of the rat model. Thus, RHA effectively ameliorates HF in the rats.

The results of metabolomic studies indicated that RHA may block the onset and progression of HF by regulating several metabolic pathways, including glycerophospholipid metabolism; phenylalanine, tyrosine, and tryptophan biosynthesis; tryptophan metabolism; and phenylalanine metabolism.

Glycerophospholipids (GPs) are the most abundant and ubiquitous phospholipids in the body. As key ingredients that form organelles and cell-cell barriers, are involved in the regulation of biological processes, such as inflammatory stress, lipid toxicity and lipid signal transduction (Mukhopadhyay and Trauner, 2023). Their metabolism is considered to be one of the key metabolic pathways associated with the phenotype of drug-induced hepatic damage (Quintas et al., 2021). Dysregulation of glycerophospholipids in the liver plays an important role in the progression mechanism of human hepatic damage (Gorden et al., 2015). In this study, we observed that the levels of metabolites such as PE (18:4 (6Z,9Z,12Z,15Z)/18:4 (6Z,9Z,12Z,15Z)), LysoPC (20:5 (5Z,8Z,11Z,14Z,17Z)/0:0) from the glycerophospholipid metabolic pathway in the CCl_4_-induced rat model were significantly altered compared with those in the controls; however, these changes were blunted in the RHA-treated group. This indicates that RHA may affect the synthesis and decomposition of glycerophospholipids by regulating the activities of key enzymes or transporters in the metabolic pathway of glycerophospholipids. It may be helpful to maintain the stability and integrity of the membrane structure, reduce the inflammatory response and oxidative stress caused by the membrane structure change, and thus prevent hepatic fibrosis (Payne et al., 2014; Paul et al., 2022).

Amino acids also play central roles in maintaining normal functions of liver. Liver is an important organ for metabolizing amino acids by producing various types of amino acid metabolism enzymes, which are involved in amino acids transamination, deamination, and transmethylation. Tryptophan and phenylalanine metabolism pathways could affect liver diseases from different perspectives (Platten et al., 2019; Lercher et al., 2020; Albillos et al., 2020). It has been shown that tryptophan could aggravate several harmful processes in the mice liver, including liver injury and fibrosis, hepatic steatosis, and production of reactive oxygen species (ROS) (Lesniak et al., 2013). Therefore, abnormal tryptophan levels in serum are therefore of importance in the pathogenesis of hepatic damage and fibrosis (Liu et al., 2019). Previous studies also have demonstrated that increased concentrations of aromatic amino acids, including phenylalanine, tyrosine and tryptophan, and methionine, are characteristic of chronic hepatic diseases, particularly cirrhosis (Williams and Lock, 2005; Kakazu et al., 2013). When the liver cells are severely damaged, it will cause amino acids metabolism disorder, especially the ratio of branched-chain amino acids to aromatic amino acids will be changed (Wernerman et al., 1999; Xu et al., 2012).

In the present study, the identified metabolites, including phenylpyruvic acid (a phenylalanine deamination product), β-tyrosine, dihydro-3-coumaric acid, trans-cinnamic acid, hydroxyphenylacetylglycine, L-dopa and phenol were mainly involved in the metabolic pathway of phenylalanine and tyrosine. Compared with the control group, a significant increase in ihydro-3-coumaric acid, trans-cinnamic acid, hydroxyphenylacetylglycine, L-dopa and phenol in the CCl_4_-treated group, while RHA reduced the levels of these metabolites in rats with hepatic fibrosis. These data suggest that RHA may play a role in alleviating hepatic fibrosis by regulating the metabolism of phenylalanine and tyrosine. However, its regulatory mechanism needs to be further verified.

Tryptophan is another essential amino acid that cannot be synthesized by human organisms and must be supplied from diets, which is necessary for protein biosynthesis. In our study, the identified metabolites, including kynurenine, N-acetyl-D-tryptophan, indole-3-acetaldehyde, 2-aminobenzoic acid, quinoline-4,8-diol, 5-hydroxyindoleacetate and kynurenic acid, all of which play significant roles in the tryptophan metabolic pathway. Notably, the quinoline-4,8-diol and 5-hydroxyindoleacetate exhibited a significant elevation in the model group, however, their concentrations diminished following RHA treatment.

Besides, lysine is one of the essential amino acids for human, which not only plays an important role in protein structures, but also in other biological processes such as structural proteins of connective tissue, calcium homeostasis, and fatty acids metabolism. However, lysine cannot be synthesized by the human body, it must be obtained from diets (Ghosh et al., 2010). In some pathological conditions, abnormal degradations of lysine may lead to the occurrence and development of diseases. For example, hepatic damage will lead to changes in lysine degradation, which in turn affects its role in the repair of cellular damage, but the molecular mechanisms still need to be further explored.

In this study, serum metabolites were assessed by non-targeted metabolomics. Non-targeted metabolomics is capable of detecting a large number of metabolites, but the identification of certain metabolites may not be accurate enough. This may lead to a biased understanding of the mechanism of RHA. Meanwhile, the massive data generated by non-targeted metabolomics requires complex bioinformatics approaches for interpretation, which may lead to subjectivity and uncertainty during data interpretation. In addition, RHA showed good anti-hepatic fibrosis effects in rats, while its pharmacodynamic and safety for humans need to be further and in-depth evaluated.

In summary, a CCl_4_-induced HF rat model was established. RHA treatment promoted the recovery of liver functions, reversed HF, and facilitated the regeneration of liver tissue in the model. Changes in small-molecule metabolites in the serum in the HF model were determined by using non-targeted metabolomics, revealing the effect of RHA on potential markers of HF and the putative underlying metabolic pathways. Overall, the presented findings indicate that RHA exerts an anti-fibrotic effect on the liver by targeting multiple signaling pathways and metabolic pathways in the liver. This study identifies RHA as a promising anti-fibrotic lead compound for further research and presents an experimental reference for the discovery of novel anti-HF agents.

## Data Availability

The original contributions presented in the study are included in the article/Supplementary Material, further inquiries can be directed to the corresponding authors.
