# Supplementary material for: Multi-target regulatory effects of rhaponticin in a rat model of hepatic fibrosis revealed by non-targeted metabolomics
Source: Front Pharmacol. 2025 Jan 14;15:1505309. doi: 10.3389/fphar.2024.1505309 (PMC11772292; doi:10.3389/fphar.2024.1505309)
Supplement: Supplementary file 1 [file DataSheet1.docx]

Supplementary Material

**Multi-target regulatory effects of rhaponticin in rat model of hepatic fibrosis revealed by non-targeted metabolomics**

Min Yang^1^, Dihua Jiang^1^, Longfei Huang^1^, Tao Zhang^1^, Wenfen Guo^1^, Wenyan Lin^1^, Jiali Zhao^1^, Yunsheng Wei^1^, Lang Peng^1^, Yong-Jia Hao,^1^* and Ying Zhou^1^*

Address: *^1^School of Pharmacy, Guizhou University of Traditional Chinese Medicine, Guiyang 550025, China*

Email: Yong-Jia Hao* – haoyongjia026@gzy.edu.cn; Ying Zhou* - yingzhou71@126.com

# Metabolomic analysis

*Quality control sample.* In order to represent the whole sample set, the quality control (QC) samples were dis+ed and tested in the same manner as the analytic samples. And they would be injected at regular intervals (every 5 samples) so as to monitor the stability of the analysis process.

*UHPLC-MS/MS analysis.* The LC-MS/MS analysis was conducted on the Thermo UHPLC-Q Exactive HF-X system equipped with an ACQUITY HSS T3 column (100 mm × 2.1 mm i.d., 1.8 μm; Waters, USA). The mobile phases are as follows: solvent A, 0.1% formic acid in the mixed solvent (water: acetonitrile = 95:5, v/v); solvent B, 0.1% formic acid in the mixed solvent (acetonitrile: isopropanol: water = 47.5:47.5:5, v/v/v). The separation gradient of +itive ion mode: 0-3 min, solvent B was increased from 0% to 20%; 3-4.5 min, solvent B was increased from 20% to 35%; 4.5-5 min, solvent B was increased from 35% to 100%; 5-6.3 min, solvent B was maintained at 100%; 6.3-6.4 min, solvent B was decreased from 100% to 0%; 6.4-8 min, solvent B was maintained at 0%. The separation gradient of -ative ion mode: 0-1.5 min, solvent B rises from 0 to 5%; 1.5-2 min, solvent B rises from 5% to 10%; 2-4.5 min, solvent B rises from 10% to 30%; 4.5-5 min, solvent B rises from 30% to 100%; 5-6.3 min, solvent B linearly maintains 100%; 6.3-6.4 min, the solvent B was decreased from 100% to 0%; 6.4-8 min, the solvent B was linearly maintained at 0%. In addition, the flow rate was 0.40 mL/min, the column temperature and injection volume were 40 ^o^C and 3 μL, respectively.

*MS conditions.* The mass spectrometric data were collected by using the Thermo UHPLC-Q Exactive HF-X Mass Spectrometer equipped with an electrospray ionization (ESI) source. The detection was carried out over a mass range of 70-1050 m/z. And the data acquisition was performed with the Data Dependent Acquisition (DDA) mode. The optimal conditions were as follows: source temperature at 425 ^o^C; sheath gas flow rate at 50 arb; Aux gas flow rate at 13 arb; ion-spray voltage floating (ISVF) at 3500 V in +itive mode and -3500 V in -ative mode, respectively. Full MS resolution and MS/MS resolution were 60000 and 7500, respectively. Normalized collision energy, 20-40-60 V rolling for MS/MS.

*Data analysis.* The pretreatment of LC/MS raw data was performed by using the Progenesis QI (Waters Corporation, Milford, USA) software. Then a three-dimensional data matrix was exported in CSV format. Both internal standard peaks and any known false +itive peaks such as noise, column bleed and derivatized reagent peaks were removed from the data matrix. Moreover, the metabolites were identified by searching databases including HMDB (http://www.hmdb.ca/) and Metlin (https://metlin.scripps.edu/). The data were analyzed through the online platform of majorbio cloud platform (cloud.majorbio.com) according to the standard procedures.

*Differential metabolites analysis.* The ‘ropls’ (version 1.6.2) was used to perform the PCA (principal component analysis) and OPLS-DA (orthogonal partial least squares-discriminant analysis). The value of VIP (variable importance in the projection) >1, *p*<0.05 were determined as significantly different metabolites. Differential metabolites were mapped into their biochemical pathways through metabolic enrichment and pathway analysis based on the KEGG database (http://www. genome.jp/kegg/). Besides, enrichment analysis was conducted by using the Python packages ‘scipy.stats’ (https://docs.scipy.org/doc/scipy/) to obtain the most relevant biological pathways.

*Statistical analysis.* The results were expressed as mean value ± standard deviation (`*x* ± s). One way ANOVA was performed by using the SPSS 26.0, and *p* < 0.05 was considered as statistically significant.

# Supplementary Tables

Table S1. The representative 25 metabolites detected in the ESI+ and ESI– modes in the control group vs. model (CCl4-induced) group comparison.

| Metabolite | Formula | M/Z | RT/min | VIP value | ESI Mode | Variation trend |
| --- | --- | --- | --- | --- | --- | --- |
| Hydroxyphenylacetylglycine | C_10_H_11_NO_4_ | 251.10 | 3.55 | 2.62 | + | ↑*** |
| D-Ornithine | C_5_H_12_N_2_O_2_ | 328.20 | 3.08 | 2.38 | + | ↑*** |
| PE(18:4(6Z,9Z,12Z,15Z)/18:4(6Z,9Z,12Z,15Z)) | C_41_H_66_NO_8_P | 377.72 | 3.65 | 2.26 | + | ↑** |
| 5-Hydroxyindoleacetate | C_10_H_9_NO_3_ | 192.07 | 3.55 | 2.21 | + | ↑** |
| PGP(20:4(8Z,11Z,14Z,17Z)/20:3(8Z,11Z,14Z)) | C_46_H_78_O_13_P_2_ | 945.49 | 4.35 | 2.14 | - | ↓*** |
| Kynurenine | C_10_H_12_N_2_O_3_ | 209.09 | 2.00 | 2.09 | + | ↑*** |
| 5-Hydroxyindoleacetic acid | C_10_H_9_NO_3_ | 192.07 | 2.01 | 2.09 | + | ↑*** |
| CDP-DG(18:3(6Z,9Z,12Z)/22:6(5Z,8E,10Z,13Z,15E,19Z)-2OH(7S, 17S)) | C_52_H_79_N_3_O_17_P_2_ | 540.76 | 2.57 | 1.91 | + | ↓* |
| 3-Dehydroquinic acid | C_7_H_10_O_6_ | 189.04 | 0.80 | 1.89 | - | ↑** |
| CDP-DG(22:6(4Z,7Z,10Z,13Z,16Z,19Z)/20:4(5Z,8Z,11Z,14Z)) | C_54_H_81_N_3_O_15_P_2_ | 537.77 | 3.96 | 1.87 | + | ↓** |
| L-Dopa | C_9_H_11_NO_4_ | 162.05 | 3.06 | 1.83 | + | ↑*** |
| Kynurenic Acid | C_10_H_7_NO_3_ | 190.05 | 2.90 | 1.77 | + | ↑*** |
| LysoPC(20:2(11Z,14Z)/0:0) | C_28_H_54_NO_7_P | 592.36 | 6.31 | 1.68 | - | ↑** |
| 3-Hydroxybenzoic Acid | C_7_H_6_O_3_ | 137.02 | 5.57 | 1.67 | - | ↑** |
| Phenylpyruvic Acid | C_9_H_8_O_3_ | 163.04 | 4.66 | 1.56 | - | ↑** |
| LysoPC(20:5(5Z,8Z,11Z,14Z,17Z)/0:0) | C_28_H_48_NO_7_P | 586.32 | 5.92 | 1.53 | - | ↑* |
| Beta-Tyrosine | C_9_H_11_NO_3_ | 164.07 | 4.07 | 1.47 | + | ↑*** |
| Quinoline-4,8-diol | C_9_H_7_NO_2_ | 162.05 | 4.60 | 1.37 | + | ↑* |
| Trans-Cinnamic Acid | C_9_H_8_O_2_ | 131.05 | 4.70 | 1.33 | + | ↑** |
| Glycerylphosphorylcholine | C_8_H_20_NO_6_P | 280.09 | 0.59 | 1.28 | + | ↑** |
| Phenol | C_6_H_6_O | 95.05 | 3.09 | 1.25 | + | ↑*** |
| Indole-3-acetaldehyde | C_10_H_9_NO | 204.07 | 4.84 | 1.21 | - | ↑* |
| GPCho(16:0/16:0) | C_40_H_80_NO_8_P | 756.55 | 7.27 | 1.18 | + | ↓* |
| 1-Linoleoyl-sn-Glycero-3-Phosphocholine | C_26_H_50_NO_7_P | 564.33 | 5.78 | 1.13 | - | ↑* |
| Dihydro-3-coumaric acid | C_9_H_10_O_3_ | 165.06 | 5.12 | 1.12 | - | ↑** |

**P*<0.05, ***P*<0.01, ****P*<0.001 vs. control group; ↓, down regulation; ↑, up-regulation; RT, retention time; VIP, variable importance in projection.

Table S2. The representative25 metabolites detected in the ESI+ and ESI– modes in the model (CCl_4_-induced) group *vs.* RHA-treated group comparison.

| Metabolite | Formula | M/Z | RT/min | VIP value | ESI Mode | Variation trend |
| --- | --- | --- | --- | --- | --- | --- |
| L-Pipecolic acid | C_6_H_11_NO_2_ | 171.11 | 2.72 | 3.31 | + | ↑* |
| LL-2,6-Diaminopimelic Acid | C_7_H_14_N_2_O_4_ | 155.08 | 1.67 | 2.48 | + | ↑* |
| Hydroxyphenylacetylglycine | C_10_H_11_NO_4_ | 251.10 | 3.55 | 2.41 | + | ↓** |
| D-Ornithine | C_5_H_12_N_2_O_2_ | 328.20 | 3.08 | 2.36 | + | ↓*** |
| 5-Hydroxyindoleacetate | C_10_H_9_NO_3_ | 192.07 | 3.55 | 2.17 | + | ↓* |
| L-Dopa | C_9_H_11_NO_4_ | 162.05 | 3.06 | 2.06 | + | ↓*** |
| PE(18:4(6Z,9Z,12Z,15Z)/18:4(6Z,9Z,12Z,15Z)) | C_41_H_66_NO_8_P | 377.72 | 3.65 | 2.00 | + | ↓* |
| L-Glutamic Acid | C_5_H_9_NO_4_ | 146.05 | 0.60 | 1.89 | - | ↑*** |
| LysoPC(20:5(5Z,8Z,11Z,14Z,17Z)/0:0) | C_28_H_48_NO_7_P | 586.32 | 5.92 | 1.89 | - | ↓* |
| 3-Dehydroquinic acid | C_7_H_10_O_6_ | 189.04 | 0.80 | 1.88 | - | ↓* |
| PE-NMe(16:0/22:5(4Z,7Z,10Z,13Z,16Z)) | C_44_H_78_NO_8_P | 824.54 | 7.04 | 1.67 | - | ↓* |
| PS(22:1(13Z)/15:0) | C_43_H_82_NO_10_P | 824.55 | 7.12 | 1.67 | - | ↓* |
| LysoPA(22:5(4Z,7Z,10Z,13Z,16Z)/0:0) | C_25_H_41_O_7_P | 526.29 | 5.99 | 1.67 | + | ↑* |
| L-Glutamate | C_5_H_9_NO_4_ | 130.05 | 0.59 | 1.66 | + | ↑*** |
| LysoPA(18:2(9Z,12Z)/0:0) | C_21_H_39_O_7_P | 433.24 | 6.14 | 1.63 | - | ↑*** |
| Quinoline-4,8-diol | C_9_H_7_NO_2_ | 162.05 | 4.60 | 1.60 | + | ↓* |
| L-Threonine | C_4_H_9_NO_3_ | 120.07 | 0.60 | 1.53 | + | ↑*** |
| LysoPC(20:3(5Z,8Z,11Z)/0:0) | C_28_H_52_NO_7_P | 590.35 | 6.16 | 1.53 | - | ↓** |
| LysoPC(16:1(9Z)/0:0) | C_24_H_48_NO_7_P | 538.32 | 6.05 | 1.37 | - | ↓** |
| Dihydro-3-coumaric acid | C_9_H_10_O_3_ | 165.06 | 5.12 | 1.26 | - | ↓** |
| PC(18:1(9Z)/18:4(6Z,9Z,12Z,15Z)) | C_44_H_78_NO_8_P | 780.55 | 5.92 | 1.25 | + | ↓** |
| Trans-Cinnamic Acid | C_9_H_8_O_2_ | 131.05 | 4.70 | 1.18 | + | ↓* |
| LysoPC(18:4(6Z,9Z,12Z,15Z)/0:0) | C_26_H_46_NO_7_P | 516.31 | 7.29 | 1.18 | + | ↓* |
| Phenol | C_6_H_6_O | 95.05 | 3.09 | 1.16 | + | ↓*** |
| L-Lysine | C_6_H_14_N_2_O_2_ | 188.14 | 0.60 | 1.09 | + | ↑** |

**P*<0.05, ***P*<0.01, ****P*<0.001 vs. control group; ↓, down regulation; ↑, up-regulation; RT, retention time; VIP, variable importance in projection.

Table S3. The 25 common metabolites in the three groups [control group vs. model (CCl_4_-induced) group; model group *vs*. RHA-treated group] showing the op+ite trend.

| Metabolite | M/Z | RT/min | Formula | A vs. B | B vs. C |
| --- | --- | --- | --- | --- | --- |
| Hydroxyphenylacetylglycine | 251.10 | 3.55 | C10H11NO4 | ↑^***^ | ↓^**^ |
| D-Ornithine | 328.20 | 3.08 | C5H12N2O2 | ↑^***^ | ↓^***^ |
| PE(18:4(6Z,9Z,12Z,15Z)/18:4(6Z,9Z,12Z,15Z)) | 377.72 | 3.65 | C41H66NO8P | ↑^**^ | ↓^*^ |
| 5-Hydroxyindoleacetate | 192.07 | 3.55 | C10H9NO3 | ↑^**^ | ↓^*^ |
| 3-Dehydroquinic acid | 189.04 | 0.80 | C7H10O6 | ↑^**^ | ↓^*^ |
| L-Dopa | 162.05 | 3.06 | C9H11NO4 | ↑^***^ | ↓^***^ |
| LysoPC(20:2(11Z,14Z)/0:0) | 592.36 | 6.31 | C28H54NO7P | ↑^**^ | ↓^*^ |
| LysoPC(20:5(5Z,8Z,11Z,14Z,17Z)/0:0) | 586.32 | 5.92 | C28H48NO7P | ↑^*^ | ↓^**^ |
| Quinoline-4,8-diol | 162.05 | 4.60 | C9H7NO2 | ↑^*^ | ↓^*^ |
| Trans-Cinnamic Acid | 131.05 | 4.70 | C9H8O2 | ↑^**^ | ↓^*^ |
| Phenol | 95.05 | 3.09 | C6H6O | ↑^***^ | ↓^***^ |
| GPCho(16:0/16:0) | 756.55 | 7.27 | C40H80NO8P | ↓^*^ | ↑^**^ |
| Dihydro-3-coumaric acid | 165.06 | 5.12 | C9H10O3 | ↑^**^ | ↓^**^ |
| Glycitein | 285.08 | 5.45 | C16H12O5 | ↑^###^ | ↓^***^ |
| Genistein | 269.05 | 5.71 | C15H10O5 | ↑^###^ | ↓^*^ |
| Quinol glucuronide | 285.06 | 3.34 | C12H14O8 | ↑^###^ | ↓^***^ |
| Interleukin-1beta (163-171) | 1037.48 | 4.5 | C39H64N12O19 | ↓^###^ | ↑^***^ |
| Taurochenodeoxycholate-3-sulfate | 288.62 | 7.12 | C26H45NO9S2 | ↓^###^ | ↑^**^ |
| 2,3,4-Trihydroxybutanoic acid | 273.08 | 3.54 | C4H8O5 | ↑^##^ | ↓^*^ |
| Urotensin-related peptide | 510.23 | 5.66 | C49H70N10O10S2 | ↓^##^ | ↑^*^ |
| Thiodiacetic acid | 192.03 | 1.12 | C4H6O4S | ↑^##^ | ↓^*^ |
| THYMOPENTIN | 662.36 | 3.72 | C30H49N9O9 | ↑^##^ | ↓^*^ |
| Valyl-prolyl-glycyl-valyl-glycine | 460.27 | 3.64 | C19H33N5O6 | ↑^##^ | ↓^*^ |
| 17-Hydroxylinolenic acid | 317.21 | 3.5 | C18H30O3 | ↑^###^ | ↓^**^ |
| S-Lactoylglutathione | 362.1 | 3.48 | C13H21N3O8S | ↑^###^ | ↓^***^ |

#*P*<0.05, ##*P*<0.01, ###*P*<0.001 vs. control group; **P*<0.05, ***P*<0.01, ****P*<0.001 *vs*. model group; ↓, downregulation; ↑, up-regulation; RT: retention time; VIP, variable importance in projection.
